# IL‐6‐specific autoantibodies among APECED and thymoma patients

**DOI:** 10.1002/iid3.109

**Published:** 2016-05-25

**Authors:** Jaanika Kärner, Maire Pihlap, Annamari Ranki, Kai Krohn, Katarina Trebusak Podkrajsek, Nina Bratanic, Tadej Battelino, Nick Willcox, Pärt Peterson, Kai Kisand

**Affiliations:** ^1^ Molecular Pathology Research Group, Institute of Biomedicine and Translational MedicineUniversity of TartuTartuEstonia; ^2^ Department of Dermatology, Allergology and Venereology, Institute of Clinical Medicine, University of Helsinki, and Skin and Allergy HospitalHelsinki University Central HospitalHelsinkiFinland; ^3^ Clinical Research Institute HUCH Ltd.HelsinkiFinland; ^4^ Unit for Special Laboratory Diagnostics, University Children's HospitalUniversity Medical CentreLjubljanaSlovenia; ^5^ Department of Pediatric Endocrinology, Diabetes and MetabolismUniversity Children's Hospital, University Medical CentreLjubljanaSlovenia; ^6^ University of Ljubljana, Medical FacultyLjubljanaSlovenia; ^7^ Department of Clinical Neurosciences, Weatherall Institute of Molecular MedicineUniversity of OxfordOxfordOX3 9DSUK

**Keywords:** Anti‐IL‐6, APECED, thymoma

## Abstract

**Introduction:**

Both autoimmune polyendocrinopathy‐candidiasis‐ectodermal dystrophy (APECED) and the rare thymoma patients with chronic mucocutaneous candidiasis (CMC) have neutralizing autoantibodies to Th17 cytokines and significant defects in production of IL‐22 and IL‐17F by their T cells. The cause of these defects is unknown. We hypothesized that they might result from autoimmunity against upstream cytokines normally responsible for generating and maintaining Th17 cells.

**Methods:**

Luciferase immunoprecipitation (LIPS) was used to screen for autoantibodies to IL‐6, IL‐1β, TGF‐β3, IL‐21, and IL‐23 in patients with APECED or thymoma. We used Western blotting to assess the conformation‐dependence of the IL‐6 autoantibodies and flow cytometric analysis of intracellular phospho‐STAT3 induction to assess IL‐6‐neutralizing capacity in IgGs isolated from patient and control sera. We also used Luminex xMAP to measure serum cytokine levels.

**Results:**

We found autoantibodies binding to conformational epitopes of IL‐6 in 19.5% of 41 patients with APECED and 12.5% of 104 with thymoma—especially in those with long disease durations. The autoantibodies were predominantly of IgG1 subclass and failed to neutralize IL‐6 activity. Notably, serum levels of the IL‐6 and IL‐17A cytokines were higher in anti‐IL‐6 seropositive than—negative APECED patients or healthy controls. We also detected autoantibody binding to IL‐23 in 27.9% of thymoma patients, resulting from cross‐recognition through the p40 subunit it shares with IL‐12.

**Conclusions:**

IL‐6 and IL‐17A elevation in these seropositive patients suggests that antibody‐binding may protect IL‐6 from degradation and prolong its half‐life in vivo.

## Introduction

Autoantibodies that block the biological functions of cytokines can predispose to certain infections. For instance, neutralizing antibodies against IFN‐γ lead to disseminated mycobacterial infections 1, 2, high‐avidity antibodies against IL‐6 cause serious staphylococcal infections 3, anti‐granulocyte macrophage colony–stimulating factor autoantibodies have been linked to cryptococcal meningitis 4, and anti‐IL‐12p70 autoantibodies to recurrent *Burkholderia gladioli* suppurative lymphadenitis 5. Moreover, autoantibodies neutralizing the T helper (Th)17 cytokines interleukin (IL)‐22, IL‐17F, and IL‐17A are associated with chronic mucocutaneous candidiasis (CMC) in most patients with the rare monogenic disease autoimmune polyendocrinopathy‐candidiasis‐ectodermal dystrophy (APECED) and also in a few patients with thymic epithelial cell neoplasia 6, 7. The obvious connections between these syndromes are aberrations in thymic epithelium 6, 8, 9, the major cell type to express the *AIRE* gene that is mutated in APECED 10, 11 and often under‐expressed in the thymoma epithelial cells 12.

Whether the Th17 cytokine‐neutralizing autoantibodies alone are sufficient to precipitate CMC is still disputable. In addition to autoantibodies, APECED and thymoma patients with CMC show severely impaired IL‐22 and IL‐17F production 6, 13, 14, 15. Interestingly, IL‐17A secretion by APECED T cells can range from almost absent in some patients to supra‐normal in some others 6, 14, 15. This led us to hypothesize that neutralizing autoantibodies to Th17‐driving cytokines (IL‐1β, IL‐6, IL‐21, IL‐23, and/or TGF‐β) 16, 17, 18 might be involved in shaping or stunting Th17 responses.

The aim of the present study was to measure autoantibodies to cytokines important for Th17 cell generation or maintenance with LIPS assays, determine their IgG isotypes, neutralizing capacity, and requirements for natural conformational epitopes.

## Materials and Methods

### Patients and controls

We studied 41 Finnish and Slovenian patients with APECED (Supporting information, Table S1, 6), 104 with thymomas, 99 of them with myasthenia gravis (MG) plus acetylcholine receptor antibodies (Supporting information, Table S2; 6, 19, 20) and healthy controls (*n* = 56; 51 ± 15.9 years old). The APECED diagnosis was confirmed by mutation analysis of *AIRE* genes and by the presence of autoantibodies to IFN‐ω and IFN‐α2. About 50% of the thymoma patients were first sampled pre‐treatment; though times and doses varied, most eventually needed corticosteroids (alternate days) ± azathioprine for their MG. Where possible, sera were stored in aliquots and only thawed immediately before use. The study was conducted in accordance with the Helsinki Declaration, with informed consent and local Ethics Committee approval.

### Autoantibody detection with LIPS

LIPS was carried out as previously reported 21, 22. IL‐6, IFN‐ω, IL‐1β, IL‐21, IL‐23A (p19), IL‐12A (p35), and IL‐12B (p40) or TGF‐β3 sequences (without signal peptide) were cloned into modified pPK‐CMV‐F4 fusion vector (PromoCell GmbH, Heidelberg; Germany) downstream of naturally secreted *Gaussia* luciferase (Gluc) that was substituted in the plasmid for Firefly luciferase. HEK293 cells were transfected with the cloned constructs, and tissue culture media containing Gluc‐fusion proteins were collected after 48 h and stored at −20°C. Serum samples were incubated with IL‐6, IFN‐ω, IL‐1β, IL‐21, IL‐23, or TGF‐β3 fusion protein solutions (2 × 10^6^ luminescence units) overnight at +4°C. Next day, Protein G agarose beads (25 µl of 4% suspension, Exalpha Biologicals, MA) were added and incubated at room temperature for 1 h in 96‐well microfilter plates (Merck Millipore, Billerica, MA) to capture antibodies and immune complexes to the beads. After washing to remove unbound fusion proteins, luciferase substrate was added (coelenterazine GAR‐2B, Targeting Systems, El Cajon, CA), and luminescence intensity (LU) measured in VICTOR X Multilabel Plate Readers (PerkinElmer Life Sciences, Waltham, MA). Results were expressed as relative units $(RU)RU=\frac{\text{LU sample}}{\text{average LU of healthy control samples}}$. The positive/negative discrimination level was set to the mean plus 3 standard deviations of the healthy control samples.

Patients with the highest binding values were selected for IL‐6 and IL‐23 blocking experiments. Briefly, the serum samples were pre‐incubated with 40 μg/ml of recombinant human (rh) IL‐6, (PeproTech EC Ltd, London, UK) or rhIFN‐γ (Miltenyi Biotec, Bergisch Gladbach, Germany). To test for IL‐23‐ blocking, the thymoma serum samples were pre‐incubated with 40 μg/ml of rhIL‐23 (PeproTech EC Ltd, London, UK), rhIL‐12 (PeproTech EC Ltd, London, UK), or rhIL‐6 (PeproTech EC Ltd, London, UK). The samples were rotated for 2 h at room temperature and centrifuged for 15 min at 16,000*g* and supernatants were transferred into new Eppendorf tubes before performing LIPS assay as above.

To render the assay IgG subclass‐specific, agarose beads coupled with streptavidin (25 µl of 4% solution, Life Technologies, Carlsbad, CA) were coated with 10 µl of biotin‐conjugated human subclass‐specific antibodies (1:100 dilution, anti‐IgG1, anti‐IgG2, anti‐IgG4, from BD Pharmingen, anti‐IgG3 from Life Technologies, Carlsbad, CA) for 1 h in microfilter plates (Merck Millipore), to capture any subclass‐specific immune complexes formed during the standard overnight pre‐incubation, before washing and readout as above. The results were expressed as RU.

### Purification of immunoglobulins

Immunoglobulins were isolated from six APECED patients’ and six control sera. Total IgG fractions were separated with fast protein liquid chromatography using Protein G Sepharose 4 Fast Flow (GE Healthcare, Biosciences, Little Chalfont, UK), eluted at pH 2.5, concentrated, and buffer‐exchanged with phosphate‐buffered saline (PBS) with iCon™ concentrator 7 ml/20 K tubes (Pierce Biotechnology, Inc., Rockford, IL). Protein concentrations were determined with Nanodrop 2000c (Thermo Scientific, Waltham, MA).

### Assay for neutralization of IL‐6 activity

We tested the APECED and control IgG fractions according to BD Phosflow™ Protocol for Human Whole Blood Samples (mild or harsh alcohol method). About 25 μl (0.6 mg/ml) of purified IgG was incubated with 25 μl (5 ng/ml) of recombinant human IL‐6 (Biolegend, San Diego, CA) at 37°C for 1 h before addition to 50 μl of fresh heparinized whole blood and incubation for a further 15 min in a 37°C water bath, and fixation for 10 min with 2 ml of pre‐warmed Lyse/Fix Buffer (BD Bioscience, Franklin Lakes, NJ). The cells were then permeabilized with pre‐chilled Perm Buffer III for 30 min on ice. Mouse anti‐human STAT3 (pY701)‐PE antibody (BD Bioscience) was used to quantify the biological effect of IL‐6 with LSR Fortessa (BD Bioscience). Results were analyzed with FCS Express 5 Plus (De Novo Software, Glendale, CA).

### Western blot

IFN‐ω and IL‐6 luciferase fusion proteins were heated at 95°C for 4 min in reducing sample buffer [3% SDS, 10% glycerol, 0.1 M dithiothreitol, 0.02% bromophenol blue and 6.25 mM Tris–HCl, pH 6.8], run in 12% SDS–PAGE and blotted onto polyvinylidene difluoride filters. After blocking, strips of the filter were incubated with patient or control sera (1:100) or rabbit anti‐luciferase antibody (1:1000 New England Biolabs, Ipswich, MA) followed by secondary antibodies (anti‐human HRP 1:10,000 and goat anti‐rabbit‐HRP 1:5000; Jackson ImmunoResearch, West Grove, PA, USA, Inc.), and visualization by enhanced chemiluminescence using the manufacturer's protocol (GE Healthcare, Little Chalfont, UK).

### Cytokine measurements

The Milliplex Magnetic Bead Panel (Millipore, Billerica, MA) was used to quantify IL‐6, IL‐17A, IL‐17F, and IL‐22 in APECED patient and control sera (stored continuously at −20°C) using the Luminex xMAP system (Luminex, Austin, TX) according to the manufacturer's protocol. Briefly, beads coupled with monoclonal antibodies to cytokines were sonicated, mixed, and diluted in bead diluent. They were incubated overnight at 4°C with serum samples, or standards or two positive controls, in 96‐well filter plates, and then with detecting antibodies for 1 h, before addition of streptavidin‐PE and analysis on a Luminex 200 instrument (Luminex) using Luminex xPONENT software (version 3.1). Positive control values fell within the expected range.

### Statistical analysis

GraphPad Software (San Diego, CA) and one‐way ANOVA with Bonferroni's Multiple Comparison test was used to assess differences between studied groups.

## Results

### APECED and thymoma patients share autoantibodies specific for IL‐6 but anti‐IL‐23 is present only in thymoma patients

**Figure 1:**
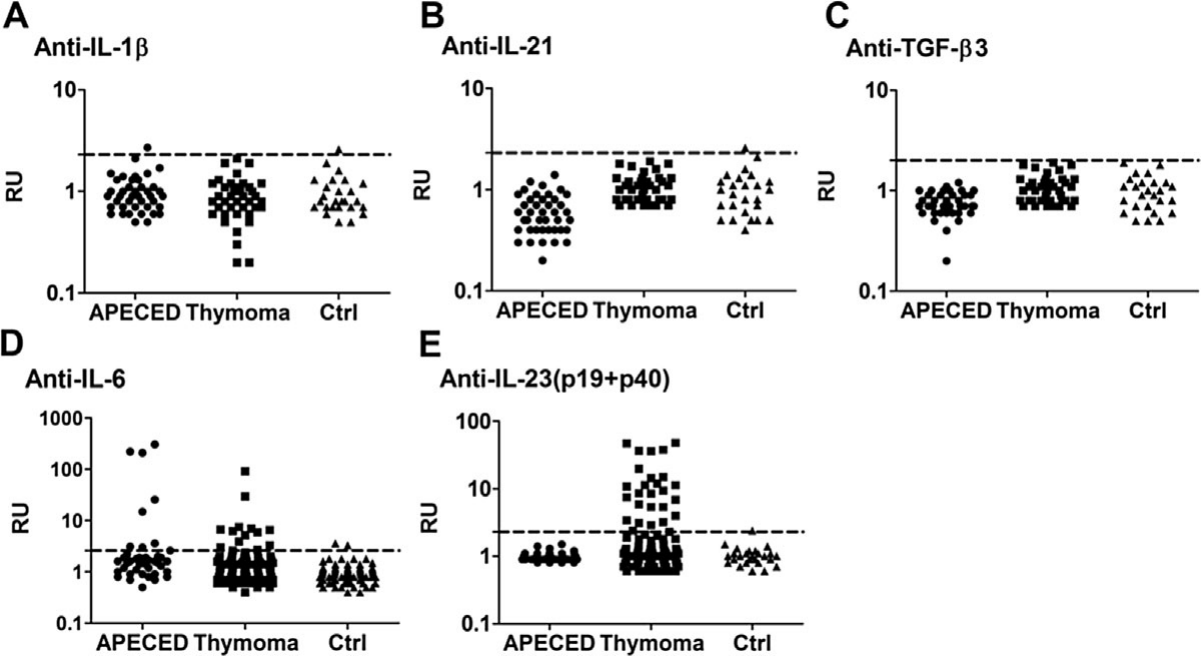
Cytokines related to Th17 maintenance and differentiation were tested with LIPS analysis for autoantibodies in APECED (41), thymoma (104) patients, and 56 healthy controls.

Sera from patients with APECED or thymomas and healthy controls were tested for autoantibodies against IL‐6, IL‐1β, IL‐21, IL‐23 (p19 + p40), or TGF‐β3 using LIPS assays that preserve the natural cytokine conformations. We could not detect any significant autoantibody binding to IL‐1β, IL‐21, and TGF‐β3 except for single borderline reactivities to IL‐1β and IL‐21 (Fig. 1A–C). But we found autoantibodies to IL‐6 in 8 of the 41 APECED (A1, A9, A18, A22, A27, A32, A34, A36) patients’ sera (19.5%, Fig. 1D; Supporting information Table S1); also in 13 (12.5%) of 104 thymoma patients (T1, T14, T23, T24, T31, T32, T34, T37, T43, T48, T50, T53, T56), though mostly at moderate levels (Fig. 1D; Supporting information Table S2). Three of the anti‐IL‐6 positive thymoma patients also had autoantibodies against IFN‐α2a, IL‐17s, and/ or IL‐22. Notably, two (patients T24 and T31) showed remarkably high IL‐17A‐ and IL‐17F‐binding and had candidiasis (Supporting information Table S2); nevertheless, these antibodies cannot be a strong predisposing factor, as two other patients with CMC and five with intercurrent *Candida* infections tested negative against IL‐6 (Table S2). Similarly, T37 had severe acne at age 25, but we found high‐level IL‐6 antibodies only >10 years later. As expected, other infections were common in the thymoma patients, many of whom were taking corticosteroids for their MG, but, again, they did not clearly co‐occur with the IL‐6 antibodies (Table S2).

Notably, two of 56 (3.6%) control sera showed moderate but consistent anti‐IL‐6 levels (Fig. 1D). Altogether, there were too few anti‐IL‐6 positive APECED and thymoma patients to justify correlations with other demographic or clinical parameters or tumor histology.

Thymoma patients are known to have autoantibodies to IL‐12 20, 23; which we found here in 91 of 104 sera (90%; Supporting information Table S2); in different cases, they recognize its p40 subunit or only the p35/p40 heterodimer (A. Meager unpublished). They also substantially inhibit Th1‐cell polarization 24. Therefore, it was not surprising to find autoantibodies to IL‐23, as it shares the p40 subunit with IL‐12. Notably, they were evident only in patients with thymoma but not in APECED. Indeed, p19 + p40 were bound by 28 of the 92 IL‐12 positive thymoma patient sera (Fig. 1E, Table S2). However, none of them showed reactivity against the IL‐23‐specific chain p19 alone (Supporting information Fig. S1). These IL‐23 autoantibodies did not correlate with CMC in thymoma patients.

**Figure 2:**
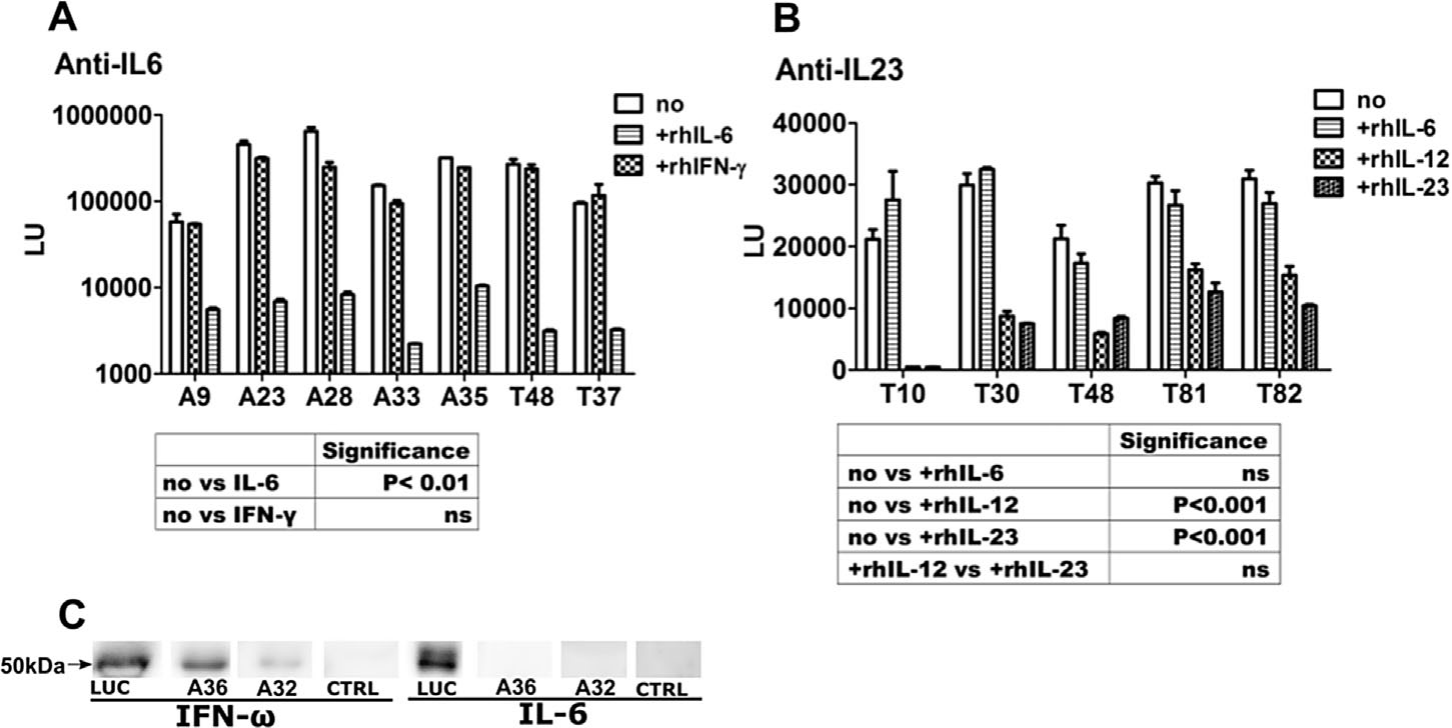
Binding specificities of IL‐6 and anti‐IL‐23 antibodies in APECED and thymoma patients. (A and B) Blocking of APECED and thymoma patients’ serum antibodies by pre‐incubation with recombinant human (rh) IL‐6, rhIFN‐γ, rhIL‐23, or rhIL‐12; no, no pre‐incubation; ns, not significant; (C) IFN‐ω and IL‐6 LUC fusion proteins were denatured in SDS buffer, separated with PAGE and analyzed by Western blot using patient control sera or anti‐luciferase (LUC) antibody. A, APECEDl; T, thymoma.

We next confirmed the specificity of the autoantibodies detected. Pre‐incubating positive APECED or thymoma patients’ sera with recombinant human (rh) IL‐6 completely blocked subsequent binding to IL‐6 in LIPS assays (*p* < 0.01), whereas a control cytokine, rhIL‐IFN‐γ, had no such effect (*p* > 0.05; Fig. 2A). Further supporting the p40‐specific cross‐reactivity between IL‐12 and IL‐23 noted above, these cytokines both strongly blocked binding by the anti‐IL‐23 antibodies (*p* < 0.0001 for each), with almost equal potency (*p* > 0.05) (Fig. 2B).

Taken together, IL‐6 was the only Th17‐driving cytokine that was recognized by autoantibodies in both APECED and thymoma patients. Moreover, these autoantibodies did not correlate with the presence or severity of CMC in either disease group, in spite of their theoretical potential to interfere with Th17 responses.

### High‐level anti‐IL‐6 autoantibodies are a late feature

We tested the available serial samples from patients with longer disease durations (Supporting information, Fig. S3). In APECED, titers against both type I IFNs and Th17 cytokines are nearly maximal at diagnosis 6, 25, and are found before onset of CMC in informative cases and even in infancy 6, 7. Moreover, they tend to decline subsequently (Supporting information, Fig. S3). Likewise, in seropositive thymoma patients, IFN‐α and/ or IL‐12 antibodies are mostly found at diagnosis, though their titers usually rise sharply if the thymoma recurs, and may vary with changes in doses of immuno‐suppressive drugs 16.

In sharp contrast, the IL‐6 autoantibodies—when present—were low initially but apparently increased over time in both patient groups, and were maximal in samples taken decades after disease onset (Supporting information, Fig. S3). In T53, they were negative at thymomectomy but positive when a recurrence was found 15 years later (not shown).

### The IL‐6 autoantibodies are mainly IgG1 and recognize conformational epitope(s)

We next modified the LIPS assay to check the IgG subclasses of the antibodies in the six strongest IL‐6‐binding sera (Table 1, three patients with APECED and three with thymoma). Reactivity was mainly in the IgG1, except in T1, where it was found in all four subclasses (Table 1).

**Table 1:** IL‐6‐specific IgG subclasses in APECED and thymoma patients

	IgG1	IgG2	IgG3	IgG4
APECED				
A23	**25**	1	**3**	1
A28	1	1	**29**	1
A35	**31**	1	1	1
Thymoma				
T1	**2**	**3**	**2**	**3**
T37	**6**	1	1	1
T48	**27**	1	1	1

The values are expressed as RU (fold changes over the average luminescence of healthy control samples). Positive values are marked bold. A, APECED patients; T, thymoma patients.

We next used Western blotting to test for recognition of conformation‐independent epitopes by the strongest APECED sera. Binding was not detected to denatured IL‐6: however, it was still strong against IFN‐ω (Fig. 2C), which we used as a positive control, as autoantibodies are known to recognize linear epitopes in type I interferons too 21. Moreover, even the strongest LIPS‐positive sera showed minimal binding to IL‐6 by ELISA (Supporting information, Fig. S2), confirming that native conformational epitopes in IL‐6 are easily disturbed.

### Autoantibodies fail to neutralize IL‐6, but correlate with higher IL‐6 levels

**Figure 3:**
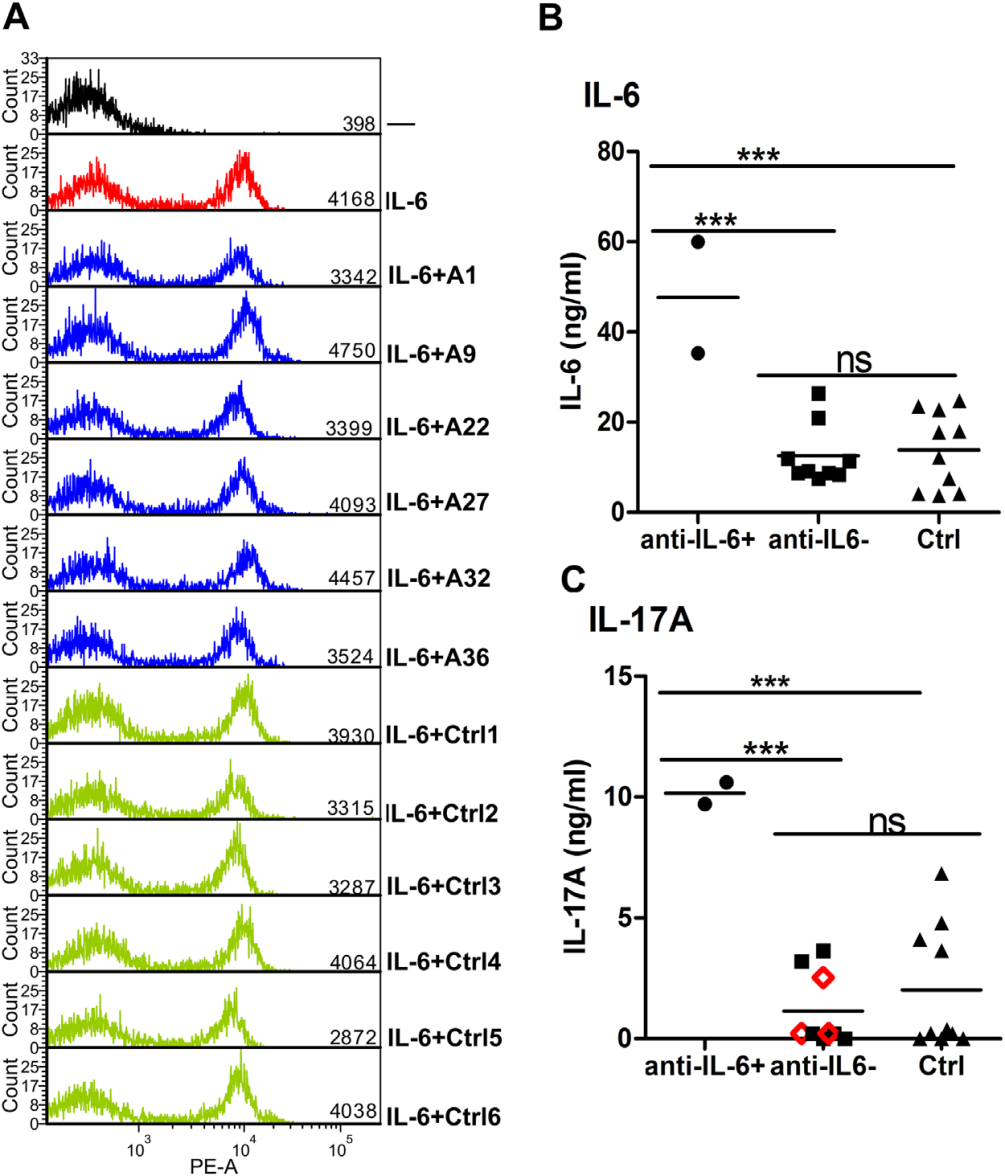
Testing for biological effects of IL‐6 antibodies (A) intracellular phospho‐STAT3 staining in cells incubated with medium alone (black), or plus IL‐6 (red) or plus IL‐6 pre‐incubated with either APECED patients’ IgG (blue) or controls’ IgG (green). Serum concentrations of IL‐6 (B) and IL‐17A (C) in APECED patients with or without IL‐6‐specific autoantibodies and in age‐matched healthy controls. APECED patients positive for anti‐IL‐17A autoantibodies are indicated with red open diamonds.

To help to understand the biological significance of these autoantibodies against IL‐6, we next tested their capacity to inhibit signaling from its receptor via phosphorylation of STAT3, which acts downstream in this pathway. FACS analysis clearly showed no inhibition by purified IgGs from six APECED patients; indeed, mean signals were even marginally higher than with the six control IgGs (3.927 vs. 3.584, *p* > 0.05; Fig. 3A). This is well in line with the rarity of staphylococcal infections in APECED patients, and argues strongly against our hypothesis that anti‐IL‐6 autoantibodies are involved in the pathogenesis of CMC in APECED patients.

Next, we studied the serum levels of IL‐6 and of the Th17 cytokines IL‐17A, IL‐17F, and IL‐22 in the available unthawed APECED and healthy control serum aliquots. IL‐17F and IL‐22 levels were below the detection limit in most of these sera, but IL‐6 and IL‐17A levels were both higher in the two anti‐IL‐6 positive patients than in the nine negatives or the ten healthy controls (*p* < 0.001, Fig. 3B and C). This finding suggests that IL‐6‐specific autoantibodies could prolong the half‐life of IL‐6 in vivo and so indirectly enhance IL‐17A production by Th17 cells.

## Discussion

In this study, we showed that among Th17‐driving cytokines, only IL‐6 was recognized by autoantibodies in some APECED and thymoma patients, and IL‐23 in some thymoma patients. Anti‐IL‐23 autoantibodies have been previously reported in two patients: one with a thymoma and the other with congenital immunodeficiency, using protein microarray and microbead‐based assays 26. Both of them were also anti‐IL‐12 positive—which strongly implicates the p40 subunit (which these cytokines share) in their cross‐recognition. That is supported by the data shown here—both the equally potent blocking of IL‐23 LIPS signals by recombinant IL‐23 and IL‐12 (Fig. 2B) and our failure to detect any reactivity toward the p19 subunit of IL‐23 (Supporting information Fig. S1). Moreover, these antibodies clearly neutralize IL‐12 in bioassays 20 and inhibit Th1‐cell polarization 24.

Like the autoantibodies against type I IFNs, Th17 cytokines and/or some organ‐specific auto‐antigens 8, those against IL‐6 were shared between APECED and thymoma patients, but were found later in the disease course and much less frequently. IL‐6 is a pleiotropic cytokine that signals through STAT3 and is evidently critical in protection against staphylococcal and fungal infections. Indeed, an autosomal dominant form of hyper‐IgE syndrome that is caused by STAT3 mutations is characterized by susceptibility to staphylococcal abscesses and CMC 27. In addition, high titer neutralizing autoantibodies to IL‐6 have previously been associated with chronic staphylococcal cellulitis and subcutaneous abscesses in one patient 3. However, studies on large numbers of healthy blood donors have revealed IL‐6‐binding reactivity in up to 9% of them and also in IgG preparations, using either RIA or ELISA in earlier studies 28, 29. 0.1% of the sera showed high binding levels and were able to neutralize the biological function of IL‐6 without any sign of disease in the respective donors 28. Disparately from these previous studies, the IL‐6 autoantibodies we detected in our APECED and thymoma patients were probably targeted to different—more conformation‐sensitive—epitopes: even adsorption to plastic abolished the binding as well as unfolding in SDS buffer. Interestingly, many of the anti‐cytokine autoantibodies in APECED are focused on conformational epitopes 21; in IL‐22, these are mostly lost when it is adsorbed to plastic for ELISAs 6. Tests carried out in solution, where its natural conformation is preserved (neutralization, LIPS, bead‐based assays), show superior sensitivity 6, 7, 22, 30. Autoantibodies to IL‐12 and IL‐23 seem to be less conformation‐dependent: many can be detected using protein arrays 26, ELISAs 20, or even immunoblotting (A. Meager, unpublished data).

Furthermore, the autoantibodies binding to IL‐6 in APECED sera did not neutralize its function (Fig. 3A). So it seems unlikely that they are involved in the pathogenesis of CMC in APECED. Conversely, IL‐6‐specific autoantibodies in systemic sclerosis have been suggested to stabilize IL‐6 in vivo as carriers, as its levels were higher in autoantibody positive patients than in negatives, and IL‐6 activity was found in the immune complexes 31. Cytokine‐stabilizing function has also been reported for anti‐IL‐2 32. Antibodies are currently thought in certain cases to increase the persistence of the cytokine in the circulation: IgG is taken up by neonatal Fc receptors (FcRn) expressed on vascular endothelium and myeloid cells. IgG is pinocytozed together with plasma and binds to FcRn in late endosomes, and is recycled back to the plasma still retaining its bound cytokine 33. Indeed, certain IL‐2/anti‐IL‐2 complexes were degraded faster in FcRn‐deficient mice 34. Notably, IgG1 has the highest affinity for nFcR 35, and was the predominant subclass in all informative samples. Whether such stabilization occurs in our patients requires further testing. However, our data indirectly suggest that the IL‐6 autoantibodies can potentiate Th17 development; the two informative anti‐IL‐6 positive patients had the highest IL‐17A levels in their sera (Fig. 3B and C). They were both anti‐IL‐17A‐negative, as were six of the nine anti‐IL‐6‐negative patients (Fig. 2C). Moreover, one of the two present anti‐IL‐6 positive patients (A35) had the highest percentage of IL‐17A‐producing T cells in our previous study (Fig. 3C from Ref. 6).

The reasons for autoimmunization against certain cytokines are not known. Autoantibodies have been detected in numerous diseases where the respective cytokine is overproduced (e.g., type I IFNs in SLE and interferonopathies) 9, 20, 36. In theory, they might modulate the disease course 37, 38. However, they rarely neutralize and their titers are much lower than those in APECED and thymoma patients 20, 30. Such low‐level IFN‐α‐binding but non‐neutralizing antibodies seemed to appear after intercurrent infections in other patient groups 20. We suspect that the infrequent IL‐6‐binding antibody responses noted here have analogous origins. That is supported by their detection only several years after diagnosis, which contrasts starkly with the same patients’ anti‐IFN‐α and IL‐22 antibodies, which are found much earlier and much more frequently, and also show intriguing early and persistent IgG4 biases 21.

## Conclusions

In conclusion, we found autoantibodies binding IL‐6 in some long‐duration APECED and thymoma patients, and autoantibodies to IL‐23 that cross‐reacted with IL‐12 in thymoma patients. These IL‐6 autoantibodies were mostly of IgG1 isotype, bound to conformational epitopes, and did not neutralize. Our data imply that they do not predispose to the candidiasis in these two syndromes. Whether they instead protect IL‐6 from degradation and prolong its half‐life in plasma, potentially enhancing its biological functions in vivo, warrants further testing.

## Authors’ Contribution

This study was conceived by KK, PP, and JK; the experiments were performed by JK, MP, and K Krohn. AR, K Krohn, KTP, NB, TB, and NW provided patient sera and clinical details. JK and KK drafted the paper, which was edited by NW and the other authors.

## Conflicts of Interest

None declared.

## Supporting information

Additional supporting information may be found in the online version of this article at the publisher's web‐site.

**Table S1**. Characteristics of APECED patients.
**Table S2**. Characteristics of thymoma patients.
**Figure S1**. Binding of IL‐23(p19+p40), IL‐23 (p19) and IL‐12(p35+40) by thymoma sera RU‐relative units.
**Figure S2**. ELISA for anti‐IL‐6.
**Figure S3**. Changes in IL‐6, IFN‐α2, IL‐17A, IL‐17F and IL‐22 specific autoantibody levels over time in APECED and thymoma patients.
