# Supplementary material for: IL‐6‐specific autoantibodies among APECED and thymoma patients
Source: Immun Inflamm Dis. 2016 May 25;4(2):235–43. doi: 10.1002/iid3.109 (PMC4879469; doi:10.1002/iid3.109)
Supplement: Supplementary file 1 — Table S1. Characteristics of APECED patients. Table S2. Characteristics of thymoma patients. Figure S1. Binding of IL‐23(p19+p40), IL‐23 (p19) and IL‐12(p35+40) by thymoma sera RU‐relative units. Figure S2. ELISA for anti‐IL‐6. Figure S3. Changes in IL‐6, IFN‐α2, IL‐17A, IL‐17F and IL‐22 specific autoantibody levels over time in APECED and thymoma patients. [file IID3-4-235-s001.docx]

**Supplementary information**

**IL-6-specific autoantibodies among** **APECED and thymoma patients**

**Short title: Anti-IL-6 autoantibodies**

Jaanika Kärner*^1^, Maire Pihlap^1^, Annamari Ranki^2^, Kai Krohn^3^, Katarina Trebusak Podkrajsek^4,6^, Nina Bratanic^5^, Tadej Battelino^5,6^, Nick Willcox^7^, Pärt Peterson^1^, Kai Kisand*^1^

^1^Institute of Biomedicine and Translational Medicine, Department of Biomedicine, Molecular Pathology Research Group, University of Tartu, Tartu, Estonia; ^2^Departments of Dermatology, Allergology and Venereology, Institute of Clinical Medicine, University of Helsinki, and Skin and Allergy Hospital, Helsinki University Central Hospital, Helsinki, Finland; ^3^Clinical Research Institute HUCH Ltd, Helsinki, Finland; ^4^Unit for Special Laboratory Diagnostics and ^5^Department of Pediatric Endocrinology, Diabetes and Metabolism, University Children's Hospital, University Medical Centre, Ljubljana, Slovenia, ^6^University of Ljubljana, Medical Faculty, Ljubljana, Slovenia; ^7^Department of Clinical Neurosciences, Weatherall Institute of Molecular Medicine, University of Oxford, OX3 9DS, UK

*Corresponding authors: [jaanika.karner@ut.ee](mailto:jaanika.karner@ut.ee), [kai.kisand@ut.ee](mailto:kai.kisand@ut.ee)

Institute of Biomedicine and Translational Medicine, Department of Biomedicine, Molecular Pathology Research Group, University of Tartu, Ravila 19, Tartu 50411, Estonia.

**Table S1** Characteristics of APECED patients

|  |  |  |  | **Autoantibodies to** | | | |
| --- | --- | --- | --- | --- | --- | --- | --- |
|  | **Age bled** | **CMC** | **IL-6** | **IL-17A** | **IL-17F** | **IL-22** | **IFN-α2** |
| A1 | 47 | + | **3.6** | 0.4 | **4.6** | **34.0** | **250.5** |
| A2 | 44 | + | 1.1 | 1.4 | **15.9** | **39.5** | **302.6** |
| A3 | 68 | + | 0.8 | 0.6 | 1.9 | **70.4** | **213.5** |
| A4 | 48 | + | 0.8 | 0.5 | 1.6 | **81.1** | **153.4** |
| A5 | 34 | + | 0.9 | 0.5 | **4.0** | **34.6** | **252.1** |
| A6 | 34 | + | 1.6 | 0.5 | **11.1** | **63.8** | **215.7** |
| A7 | 58 | + | 0.9 | 0.5 | **34.0** | **87.6** | **439.8** |
| A8 | 34 | + | 1.4 | **17.1** | **3.2** | **54.9** | **443.8** |
| A9 | 27 | + | **25.6** | 1.9 | **3.2** | **77.2** | **449.7** |
| A10 | 48 | + | 1.0 | 0.6 | **3.1** | **55.2** | **183.3** |
| A11 | 52 | + | 1.2 | 0.5 | 2.3 | **15.2** | **194.3** |
| A12 | 47 | + | 1.9 | **423.5** | **14.1** | **41.1** | **142.1** |
| A13 | 45 | + | 1.3 | **7.5** | **24.1** | **106.8** | **193.5** |
| A14 | 19 | ‒ | 1.5 | 1.4 | **8.7** | **12.0** | **400.4** |
| A15 | 19 | + | 0.8 | 1.3 | **21.8** | **75.2** | **311.7** |
| A16 | 54 | + | 1.9 | 0.8 | 0.9 | **32.8** | **437.5** |
| A17 | 20 | + | 1.4 | **765.6** | **44.7** | **45.7** | **282.5** |
| A18 | 56 | + | **3.1** | **11.9** | **5.8** | **45.3** | **45.4** |
| A19 | 60 | + | 1.6 | **502.8** | **11.6** | **136.8** | **170.3** |
| A20 | 53 | + | 1.2 | **13.4** | **4.8** | **74.1** | **173.5** |
| A21 | 37 | + | 2.2 | **3.3** | **14.9** | **199.5** | **286.9** |
| A22 | 39 | + | **220.7** | 0.8 | 0.8 | **37.1** | **279.1** |
| A23 | 73 | + | 1.5 | 0.8 | 2.3 | **55.5** | **436.2** |
| A24 | 46 | + | 1.6 | 0.5 | **3.3** | **11.8** | **419.4** |
| A25 | 46 | + | 1.6 | **10.6** | **7.3** | **86.7** | **222.2** |
| A26 | 22 | + | 2.6 | 2.5 | **22.7** | **136.6** | **353.4** |
| A27 | 58 | + | **304.9** | **4.9** | **3.4** | **67.6** | **235.2** |
| A28 | 7 | ‒ | 1.9 | 0.7 | **23.8** | **122.6** | **6.7** |
| A29 | 14 | + | 0.7 | **343.9** | **50.6** | **263.8** | **276.0** |
| A30 | 12 | + | 0.9 | **32.2** | **29.5** | **260.6** | **349.8** |
| A31 | 21 | + | 0.5 | 0.7 | 0.8 | **77.4** | **149.0** |
| A32 | 16 | + | **14.9** | 0.7 | **19.2** | **59.1** | **278.3** |
| A33 | 25 | + | 1.7 | **145.4** | **5.9** | **59.1** | **280.9** |
| A34 | 23 | + | **209.3** | 1.3 | **75.3** | **80.6** | **242.4** |
| A35 | 27 | + | 1.9 | **73.2** | **52.6** | **61.4** | **175.5** |
| A36 | 23 | + | **3.0** | 0.9 | **19.9** | **72.2** | **139.4** |
| A37 | 34 | + | 1.6 | 1.1 | **16.5** | **140.2** | **133.2** |
| A38 | 21 | + | 1.8 | **3.0** | **27.0** | **103.1** | **92.3** |
| A39 | 9 | + | 0.7 | **8.4** | **16.5** | **81.2** | **103.6** |
| A40 | 4 | + | 2.0 | 0.8 | **24.5** | **61.3** | **73.6** |
| A41 | 15 | + | 1.0 | **6.9** | **6.3** | **80.6** | **101.1** |

**Table S2.** Characteristics of thymoma patients

|  |  |  |  |  |  | **Autoantibodies to (RU; fold change)** | | | | | |  |  |
| --- | --- | --- | --- | --- | --- | --- | --- | --- | --- | --- | --- | --- | --- |
|  | **Sex** | **MG** | ***Candi*** | **Tumor recurred** | **IL-6** | **IL-12** | **IL-23** | **IL-17A** | **IL-17F** | **IL-22** | **IFN-α** | **Clinical features ^a^** |  |
| T1 | F | + | ‒ | no Tx | **5.2** | **284.8** | **10.8** | 0.5 | 0.1 | 0.6 | 0.4 | neuromyotonia; alopecia/ erythema/ skin "like GVH", chest infections; † 73 |  |
| T2 | M | + | ‒ |  | 0.9 | **5.6** | 1.2 | 0.3 | 0.2 | 0.8 | **50.9** |  |  |
| T3 | M | + | ‒ |  | 1.5 | **149.1** | 0.7 | 0.3 | 0.2 | 0.8 | **23.0** |  |  |
| T4 | M | + | ‒ |  | 1.6 | **2.4** | 0.8 | 0.5 | 0.1 | 0.5 | 0.5 |  |  |
| T5 | M | + | ‒ | + | 2.0 | **39.5** | 0.8 | 0.7 | 0.3 | **11.3** | **38.3** | fungal lung granulomas; ulcerative colitis, †~50 |  |
| T6 | M | + | ‒ |  | 1.6 | 1.3 | 0.9 | 0.7 | 0.1 | 0.5 | **29.2** | nephrotic syndrome |  |
| T7 | F | + | ‒ |  | 1.2 | **176.1** | **12.0** | 1.3 | 0.5 | 0.6 | 0.5 | urinary infections, goitre |  |
| T8 | M | + | ‒ |  | 0.7 | **284.8** | **15.0** | 1.2 | 0.2 | 1.7 | **47.6** |  |  |
| T9 | F | + | ‒ |  | 1.1 | **63.3** | **2.9** | 1.0 | 0.2 | 0.4 | **25.9** |  |  |
| T10 | F | + | ‒ |  | 2.4 | **342.7** | **36.0** | 0.4 | 0.1 | 0.6 | **65.7** | vitiligo; asplenia; neuromyotonia; hypothyroid; SLE; urticaria |  |
| T11 | F | + | ‒ |  | 1.4 | **55.3** | 1.9 | 0.5 | 0.2 | 0.7 | **26.3** | Crohn's disease |  |
| T12 | F | + | ‒ |  | 1.2 | **2.2** | 1.0 | 1.2 | 0.2 | 0.5 | **52.0** |  |  |
| T13 | M | + | ‒ |  | 1.3 | **2.1** | 1.0 | 0.5 | 0.1 | **23.6** | **7.1** |  |  |
| T14 | F | ‒ | ‒ |  | **3.3** | **3.0** | 1.4 | 0.4 | 0.1 | 0.8 | 0.5 | hypogammaglobulinemia, adrenal insuffiency, urticaria, tongue lichen planus |  |
| T15 | M | + | ‒ |  | 1.2 | **2.1** | 0.9 | 0.9 | 0.2 | 0.5 | **38.0** |  |  |
| T16 | M | + | ‒ |  | 1.2 | **5.2** | 0.6 | 0.5 | 0.3 | 0.7 | **28.7** | obstipation |  |
| T17 | F | + | ‒ |  | 0.6 | **320.5** | **11.3** | 1.3 | 0.1 | 0.4 | **4.5** | Carcinomas of breast duct and nasal septum |  |
| T18 | F | + | ‒ |  | 0.7 | **3.6** | 1.0 | 0.4 | 0.1 | 0.6 | **34.9** | RA; hypothyroid, † ~50 |  |
| T19 | M | + | ‒ |  | 1.0 | **160.1** | **3.4** | 0.3 | 0.1 | 0.6 | **36.5** | Chronic Obstructive Pulmonary Disease |  |
| T20 | F | + | ‒ | + | 1.4 | **272.2** | 1.0 | 0.5 | 0.1 | 0.6 | **13.3** | age 35 sinusitis/ respiratory infections, progressive multi-focal leukoencephalopathy, lung *Aspergillus*, *H. zoster* † ~43 |  |
| T21 | F | + | ‒ | + | 1.6 | 1.7 | 0.8 | 0.6 | 0.1 | 1.0 | **19.7** | Chronic Obstructive Pulmonary Disease |  |
| T22 | M | + | ‒ |  | 0.7 | **208.4** | 0.8 | 0.4 | 0.1 | 1.0 | **20.1** |  |  |
| T23 | M | + | ‒ |  | **6.3** | **4.6** | 1.0 | 0.2 | 0.1 | 0.4 | 1.7 |  |  |
| T24 | F | + | + |  | **6.6** | n.d | n.d | **100.2** | 0.4 | **103.2** | **29.6** | oral *Candida* |  |
| T25 | F | + | ‒ |  | 0.6 | **122.0** | 1.3 | 0.4 | 0.1 | 0.9 | **19.4** | alopecia, sinusitis/ respiratory infections |  |
| T26 | M | + | ‒ |  | 0.8 | **3.8** | 0.9 | 0.2 | 0.1 | 0.5 | **16.6** |  |  |
| T27 | M | + | ‒ | + | 1.9 | **238.7** | 1.1 | 0.3 | 0.1 | 1.3 | **59.8** | T1D, alopecia, vitiligo, nail dystrophy, thyroid atrophy, stiff man syndrome, † 65 |  |
| T28 | M | + | ‒ |  | 1.0 | **263.4** | **11.4** | 0.3 | 0.1 | 0.7 | **50.4** |  |  |
| T29 | M | + | ‒ |  | 0.6 | **4.3** | 0.8 | 0.4 | 0.6 | 1.6 | **20.9** |  |  |
| T30 | M | + | ‒ |  | 1.2 | **305.2** | **48.0** | 0.4 | 0.1 | 0.7 | **61.8** | brain abscess (on steroids); †~70 |  |
| T31 | F | + | + ^b^ |  | **3.0** | **202.3** | 0.8 | **95.2** | **11.4** | **71.0** | **24.4** | goitre, CMC; † 73 |  |
| T32 | M | ‒ | ‒ |  | **2.7** | **4.4** | **2.0** | 0.5 | 0.2 | **5.9** | 0.2 | chest infection→X-ray finding |  |
| T33 | F | + | ‒ |  | 1.6 | **138.1** | 1.0 | 1.0 | 0.2 | 0.5 | 1.3 | alopecia, red cell aplasia |  |
| T34 | M | + | ‒ |  | **6.7** | **6.4** | 1.5 | 0.7 | 0.1 | 0.7 | **15.5** | AIDS-like syndrome" but HIV-negative, incl CMV,  B lymphoma & *Pneumocystis* [on azathioprine], † 48 |  |
| T35 | F | + | ‒ |  | 2.0 | 1.7 | 0.7 | 0.4 | 0.6 | 1.2 | 0.5 | neuromyotonia |  |
| T36 | F | + | ‒ | + | 1.9 | **144.7** | **9.4** | 2.0 | 0.1 | **68.2** | **13.0** | † ~55 |  |
| T37 | M | + | ‒ |  | **29.7** | **4.7** | 1.8 | **3.1** | 0.2 | 1.3 | **43.6** | severe acne; cheloid scar |  |
| T38 | M | + | ‒ |  | 1.2 | **2.1** | 0.8 | 0.6 | 0.2 | 0.5 | **25.6** |  |  |
| T39 | M | + | ‒ |  | 1.5 | **242.9** | **36.7** | 0.6 | 0.3 | 0.5 | 0.3 |  |  |
| T40 | F | + | ‒ |  | 1.5 | 0.8 | 0.7 | 0.3 | 0.2 | 1.4 | **17.4** |  |  |
| T41 | M | + | ‒ |  | 1.5 | **218.3** | **8.5** | 0.4 | 0.1 | 0.7 | **53.2** |  |  |
| T42 | F | + | ‒ |  | 1.3 | **248.2** | **7.5** | 0.4 | 0.2 | 0.8 | **28.1** |  |  |
| T43 | F | + | ‒ |  | **7.0** | **2.2** | 1.3 | 0.2 | 0.2 | 0.7 | **28.8** |  |  |
| T44 | F | + | + ^b^ | + | 1.1 | **4.3** | 0.7 | 1.4 | 0.2 | 1.3 | 0.6 | CMV retinitis, CMC, †47 |  |
| T45 | M | + | ‒ |  | 0.8 | 1.1 | 1.0 | 0.3 | 0.1 | 1.2 | **42.2** | asthma, ezcema |  |
| T46 | M | + | ‒ |  | 1.3 | **5.6** | 0.9 | 0.6 | 0.1 | 0.7 | **20.3** | myositis; chest infections; pseudo-membranous colitis |  |
| T47 | F | + | ‒ |  | 1.3 | **3.1** | 1.1 | 0.4 | 0.2 | **41.0** | **45.2** | † septicemia ~84 |  |
| T48 | F | + | ‒ |  | **92.5** | **575.7** | **37.9** | 0.3 | 0.2 | 1.5 | **59.9** | septicemia on steroids,  *H. zoster* |  |
| T49 | F | + | ‒ |  | 1.4 | **2.8** | 1.1 | 0.6 | 0.2 | **39.5** | **53.8** | T1D, nephrotic syndrome |  |
| T50 | M | + | ‒ |  | **3.9** | **204.7** | **8.6** | 0.5 | 0.2 | 0.4 | 0.3 |  |  |
| T51 | F | + | ‒ |  | 1.2 | **12.4** | 0.9 | 0.5 | 0.1 | 0.9 | **46.3** | alopecia; polymyositis; neuromyotonia, † 75 |  |
| T52 | M | + | ‒ |  | 1.4 | 1.3 | 0.9 | 0.4 | 0.2 | 0.9 | **74.2** |  |  |
| T53 | F | + | ‒ | + | **7.5** | **302.4** | 1.0 | 0.3 | 0.2 | 0.5 | **9.0** |  |  |
| T54 | F | + | ‒ |  | 0.7 | **11.3** | 0.6 | **4.0** | 0.2 | **183.0** | 0.8 |  |  |
| T55 | M | + | ‒ |  | 0.7 | **2.5** | 0.6 | 0.4 | 0.1 | 0.6 | **18.0** | "infections" |  |
| T56 | F | ‒ | + | no Tx | **5.9** | **2.4** | 0.9 | 0.3 | 0.1 | 1.0 | 0.5 | myositis, *H. zoster*, oral *Candida* |  |
| T57 | M | + | ‒ |  | 1.3 | **260.5** | **2.1** | 0.3 | 0.1 | 0.8 | 1.1 |  |  |
| T58 | M | + | ‒ |  | 0.8 | **2.1** | 0.6 | 0.3 | 0.1 | 0.7 | 0.5 |  |  |
| T59 | F | + | ‒ |  | 1.7 | **99.7** | **5.4** | 0.3 | 0.1 | 0.9 | **63.6** | *H. zoster*; respiratory infections |  |
| T60 | M | + | ‒ |  | 0.9 | **205.5** | 0.6 | 0.4 | 0.2 | **34.5** | **37.6** |  |  |
| T61 | M | + | ‒ |  | 0.7 | 1.0 | 0.8 | 0.4 | 0.1 | 0.7 | **23.0** |  |  |
| T62 | M | + | ‒ |  | 1.2 | **304.8** | **3.1** | 0.3 | 0.1 | 0.8 | 1.0 | nephrotic syndrome;†~80 |  |
| T63 | F | + | ‒ |  | 0.9 | **202.2** | **6.9** | 0.4 | 0.1 | 0.9 | **16.9** |  |  |
| T64 | M | + | ‒ |  | 1.7 | **164.8** | **5.4** | 0.4 | 0.1 | 0.9 | **51.1** | alopecia;  † 42 myocardial infarct |  |
| T65 | F | + | ‒ |  | 1.0 | **23.6** | 0.8 | 0.3 | 0.1 | 0.7 | 0.8 |  |  |
| T66 | F | ‒ | ‒ |  | 1.4 | **2.2** | 1.1 | 0.2 | 0.1 | 0.4 | **3.9** | hypogammaglobulinemia |  |
| T67 | M | + | ‒ |  | 0.8 | **4.7** | 1.2 | 0.4 | 0.2 | 1.1 | **53.4** |  |  |
| T68 | F | + | ‒ |  | 1.2 | **3.1** | 1.0 | 0.6 | 0.3 | 0.9 | **68.8** | optic neuritis |  |
| T69 | M | + | ‒ |  | 1.2 | **1.9** | 0.6 | 0.3 | 0.1 | 0.5 | **60.3** |  |  |
| T70 | M | ‒ | ‒ |  | 0.6 | **210.4** | 0.8 | 0.8 | 0.1 | 0.7 | **3.7** | systemic sclerosis |  |
| T71 | M | + | ‒ |  | 2.1 | **115.4** | **5.9** | 0.5 | 0.1 | 0.8 | **59.2** | Behçet's; incipient respiratory failure; T2D. bladder polyps, diverticulitis;“several chest infections" |  |
| T72 | M |  |  |  | 0.7 | **3.3** | 0.7 | 0.6 | 0.1 | 1.3 | **30.3** | bronchiectasis, Parkinson's |  |
| T73 | F | + | + |  | 0.8 | **474.3** | 0.9 | 0.5 | 0.1 | 0.9 | **34.9** | oral *Candida* |  |
| T74 | F | ‒ | ‒ |  | 1.1 | **60.7** | **14.4** | 1.4 | 0.1 | **64.6** | **16.8** | neuromyotonia |  |
| T75 | M | + | + | + | 0.7 | **194.7** | **2.4** | 0.6 | 0.7 | 1.0 | **38.9** | lung fibrosis, candidiasis at esophagoscopy, *Listeria mono-cytogenes* meningitis, † ~53 |  |
| T76 | M | + | ‒ |  | 0.7 | **144.7** | 0.7 | 0.5 | 0.1 | 0.6 | **53.7** |  |  |
| T77 | F | + | ‒ |  | 0.6 | **449.7** | 1.2 | 0.3 | 0.1 | 0.6 | 0.1 | hyperthyroid |  |
| T78 | M | + | ‒ |  | 1.8 | **314.7** | 1.8 | 1.0 | 0.1 | 0.9 | 0.2 | † 37 suddenly post-‘flu’ |  |
| T79 | F | + | + |  | 0.6 | **11.4** | 1.1 | 0.3 | 0.1 | 0.5 | **18.3** | intertrigo |  |
| T80 | F | + | ‒ |  | 0.7 | **237.8** | 1.7 | 1.3 | 0.1 | 0.7 | **12.6** |  |  |
| T81 | M | + | ‒ |  | 0.6 | **310.8** | **19.8** | 0.4 | 0.2 | 0.7 | **5.3** | neuromyotonia, †74 |  |
| T82 | F | + | ‒ |  | 2.2 | **507.4** | **47.3** | 0.6 | 0.3 | 0.8 | **16.9** |  |  |
| T83 | F | + | ‒ |  | 0.4 | **3.4** | **4.0** | 0.8 | 0.3 | 0.9 | **20.5** |  |  |
| T84 | F | + | ‒ |  | 0.5 | **230.8** | 1.0 | 0.3 | 0.2 | 0.9 | 2.1 | breast fibroadenoma |  |
| T85 | F | + | ‒ |  | 0.5 | **139.6** | 1.2 | 0.3 | 0.1 | 1.6 | 0.2 |  |  |
| T86 | F | + | ‒ | + | 0.6 | 1.1 | 1.2 | **45.5** | 0.1 | 1.4 | **22.2** | septicemia, neutropenia, poly-arthritis, *Candida* oropharynx, Cryptococcal meningitis, †45 |  |
| T87 | F | + | ‒ |  | 0.6 | **2.9** | 0.8 | 0.3 | 0.1 | 0.5 | **22.0** |  |  |
| T88 | F | + | ‒ |  | 1.1 | 0.8 | 0.8 | **89.5** | **5.6** | **52.1** | **69.6** | hypothyroid |  |
| T89 | M | **+** | **+ ^b^** | + | 2.0 | **225.2** | 0.6 | 0.7 | 0.1 | 0.6 | 0.3 | neuromyotonia: CMC, |  |
|  |  |  |  |  |  |  |  |  |  |  |  | *H. zoster*; † 60 |  |
| T90 | F | + | ‒ |  | 0.7 | **16.2** | 1.0 | 0.5 | 0.2 | 0.8 | **10.3** | papillary thyroid carcinoma |  |
| T91 | F | + | ‒ |  | 0.7 | **152.6** | 1.4 | **3.2** | 0.2 | **45.0** | **19.2** | hyperthyroid nodule |  |
| T92 | F | + | + | + | 1.8 | **365.8** | 0.9 | 0.3 | 0.1 | 0.5 | 0.2 | red blood cell aplasia, oral *Candida*, *H. zoster*; †64 |  |
| T93 | M | + | ‒ |  | 0.7 | **344.4** | 1.3 | 0.3 | 0.1 | 0.6 | **19.5** |  |  |
| T94 | F | + | ‒ |  | 1.0 | **20.5** | **3.2** | 0.5 | 0.1 | 0.7 | **66.9** |  |  |
| T95 | M | + | ‒ |  | 0.7 | 1.2 | 0.8 | 0.4 | 0.1 | 0.6 | 0.7 |  |  |
| T96 | F | + | ‒ |  | 0.6 | **63.8** | 1.0 | 1.5 | 0.2 | **29.2** | **63.7** |  |  |
| T97 | F | + | ‒ |  | 1.0 | **3.8** | 1.1 | 0.3 | 0.2 | 0.0 | 0.0 |  |  |
| T98 | F | + | ‒ |  | 0.7 | 0.8 | 0.7 | 0.7 | 0.2 | 0.8 | **30.8** |  |  |
| T99 | M | + | ‒ |  | 0.9 | **331.6** | 0.6 | 0.6 | 0.1 | 0.8 | **66.2** |  |  |
| T100 | F | + | ‒ |  | 0.6 | **3.1** | 1.2 | 0.5 | 0.2 | 0.3 | 0.5 | skin tumors, skin SLE |  |
| T101 | M | ‒ | ‒ |  | 0.7 | **91.1** | 1.8 | 0.9 | 0.5 | 0.9 | **76.3** | Chronic Obstructive Pulmonary Disease |  |
| T102 | F | + | ‒ |  | 0.5 | **2.9** | 2.3 | 0.3 | 0.1 | 0.8 | **59.1** |  |  |
| T103 | M | + | ‒ |  | 2.6 | 1.2 | 1.2 | **7.6** | 0.1 | 1.4 | 1.2 |  |  |
| T104 | M | + | ‒ |  | 0.6 | **129.2** | 0.7 | 0.5 | 0.1 | 0.6 | 0.9 |  |  |

^a^ clinical features are in order of onset

**^b^** Yellow/green shading indicates true CMC, and yellow = inter-current *Candida* infections possibly related in part to immuno-suppressive drug treatments

Values **in bold** considered positive; where serial samples were tested, the results are those for the last date.

Nearly all of the thymomas were removed at thoracotomy, except for T1 and T56. Most patients were given corticosteroids (alternate day) ± azathioprine at varying stages and doses for their MG *et al*, and most with thymoma recurrences had chemo- ± radio-therapy. no Tx, no thymomectomy

Ca, carcinoma; COPD, Chronic Obstructive Pulmonary Disease; T1D or T2D types 1 or 2 diabetes; nd, not done


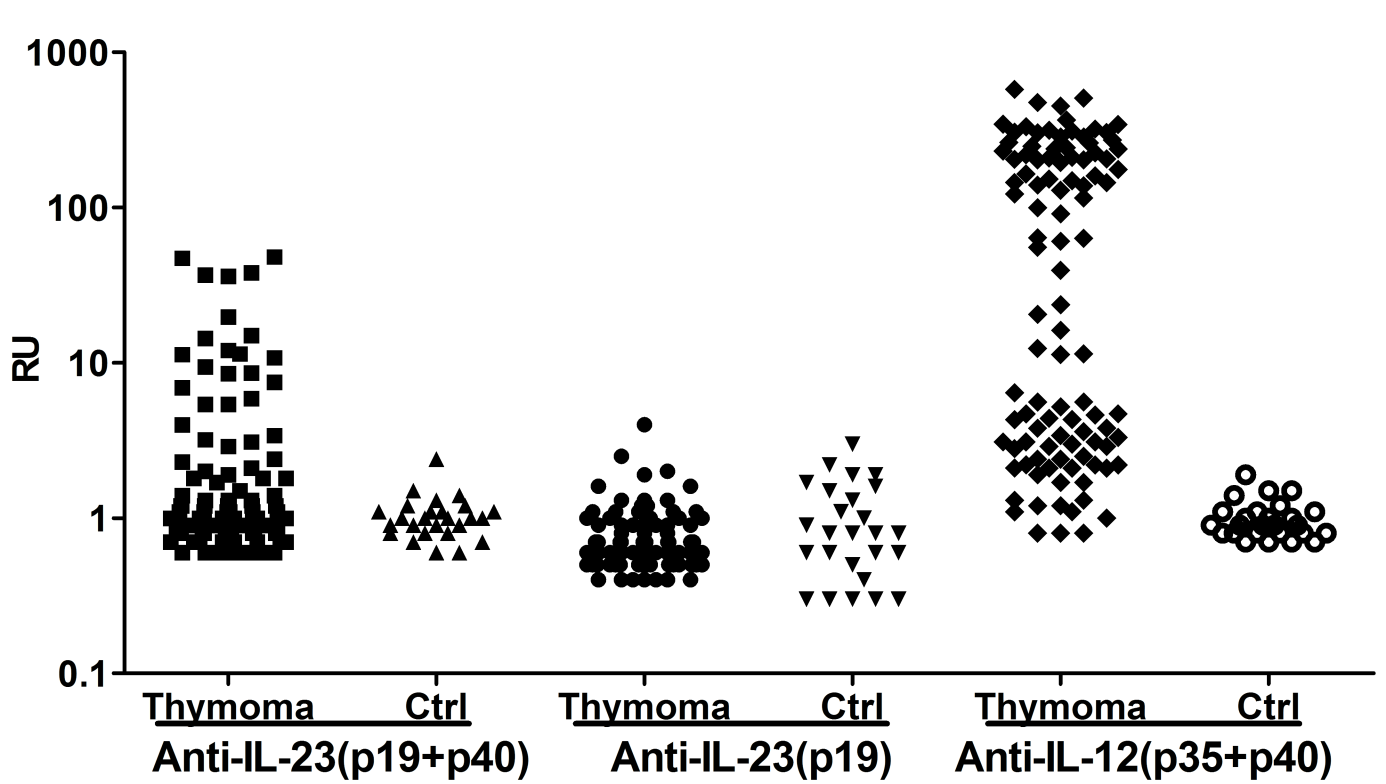


**Fig. S1** Binding of IL-23(p19+p40), IL-23 (p19) and IL-12(p35+40) by thymoma sera

RU-relative units.


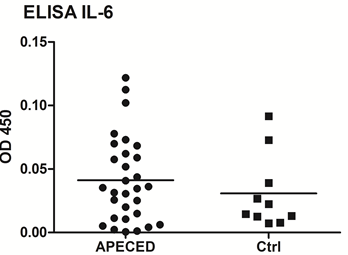


**Fig. S2** ELISA for anti-IL-6

30 APECED and 10 control sera were tested. 96 well half-area ELISA plates were coated with 30µl of IL-6 (0.75 µg/ml) (Immuno-tools, Germany) in sodium potassium carbonate-bicarbonate buffer (Sigma-Aldrich Corporation, St. Louis, Missouri, United States). After overnight incubation at +4 ºC the plates were blocked for 1 hour with 1% BSA (Sigma-Aldrich Corporation), washed with a washing buffer (Sigma-Aldrich Corporation) before addition of 30 µl of serum dilution (1:500) in 0.5% BSA/PBS buffer. After overnight incubation at +4 ºC, the plates were again washed and peroxidase-conjugated goat anti-human IgG Fc (Jackson ImmunoResearch) was added for 1 hour, followed by washing and adding the TMB-ELISA substrate. The reaction was stopped with 2M H_2_SO_4_ solution and the plates were read for optical density with the Multiskan FC ELISA reader.


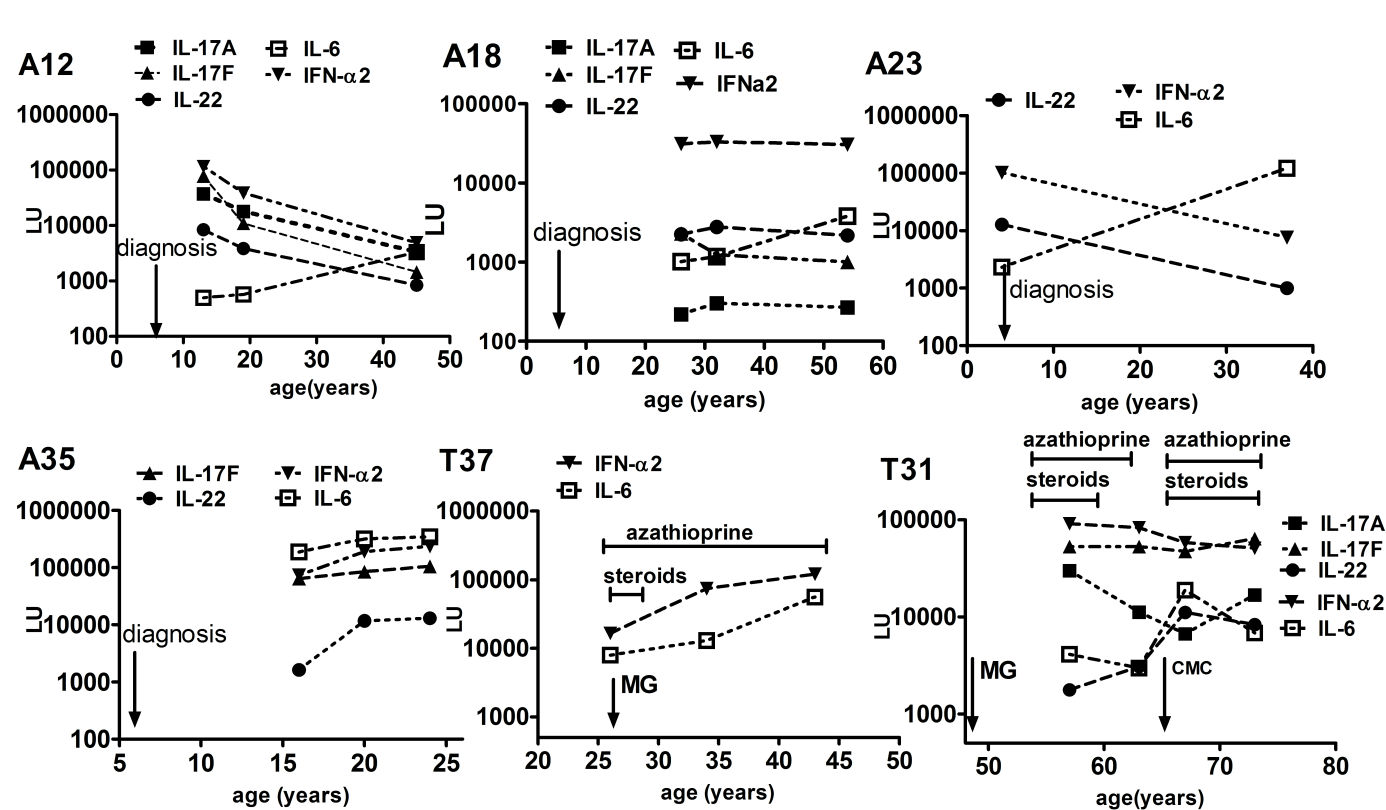
**Fig.S3** Changes in IL-6, IFN-α2, IL-17A, IL-17F and IL-22 specific autoantibody levels over time in APECED and thymoma patients. We detected no antibodies against Th17-related cytokines in patient T37.

A - APECED, T – thymoma, LU-luminescence
